# Evaluating a Digitally Delivered, Multi-Modal Intervention for Parents of Children with Type 1 Diabetes: A Proof-of-Concept Study

**DOI:** 10.3390/children11091114

**Published:** 2024-09-12

**Authors:** Tricia S. Tang, Niloufar Sharif, Crystal Ng, Logan McLean, Gerri Klein, Shazhan Amed

**Affiliations:** 1 Department of Medicine, Faculty of Medicine, University of British Columbia, Vancouver, BC V5Z 1M9, Canada; 2 T1D Huddle Gordon & Leslie Diamond Health Care Centre, Vancouver, BC V5Z 1M9, Canada; logan.a.mclean@gmail.com; 3 Faculty of Education, School of Kinesiology, University of British Columbia, Vancouver, BC V6T 1Z1, Canada; nshariif@student.ubc.ca; 4 Department of Pediatrics, Faculty of Medicine, University of British Columbia, Vancouver, BC V6H 0B3, Canada; crystal.ng@cw.bc.ca (C.N.); samed@cw.bc.ca (S.A.); 5 BC Children’s Hospital, Vancouver, BC V6H 3N1, Canada; 6 BC Diabetes, Vancouver, BC V5Y 3W2, Canada; gklein@bcdiabetes.ca

**Keywords:** digital interventions, parents, peer support, type 1 diabetes

## Abstract

**Background/Objectives:** We examined the feasibility, acceptability, and potential mental health impact of a digital peer support intervention involving videoconferencing and text-based support for parents of school-aged children living with T1D and analyzed posts exchanged by parents on a texting platform. **Methods:** Eighteen parents were recruited for Huddle4Parents, a 4-month digital intervention that involved four synchronous group-based Zoom sessions coupled with an asynchronous 24/7 peer support texting room. Primary outcomes were feasibility (i.e., ability to recruit *n* = 20 parents and retain at least 75%) and acceptability (i.e., satisfaction ratings of “good” to “very good”). Baseline and 4-month assessments also measured diabetes distress, quality of life, and perceived support. A content analysis of text exchanges was also performed. **Results:** All 15 parents who completed the intervention attended at least one Huddle and posted at least one message on the 24/7 peer support room. The retention rate was 83%, with 100% indicating that they would “definitely” or “probably yes” recommend both platforms to other parents. They also rated the topics, facilitator, and overall Huddles as “good” to “excellent.” No changes were observed for psychosocial endpoints. Of the 1084 texts posted, core support themes included the following: (1) dealing with technology and devices; (2) seeking and providing emotional support; (3) managing T1D in the school setting; and (4) exchanging tips and strategies. **Conclusions:** Huddle4Parents, a digital T1D caregiver intervention offering synchronous and asynchronous support, is feasible based on recruitment, participation, and attrition rates and acceptable as demonstrated by engagement and satisfaction ratings for the Huddles and 24/7 peer support room.

## 1. Introduction

Parents are primarily responsible for the day-to-day management of children with type 1 diabetes (T1D), as the latter have not developed the cognitive, emotional, and behavioral skills needed to meet the self-care demands [1,2]. Thus, diabetes management significantly impacts parents, not only in terms of their physical, psychological, and emotional well-being but also in their relationships, personal decisions, financial stability, and daily routines [3,4]. The burden and complexity of diabetes management and fear of short- and long-term complications are associated with greater diabetes distress (DD) and depressive symptoms in parents of children with T1D [4,5,6]. According to a systematic review of 34 studies, 19% of parents reported DD one to four years following T1D diagnosis [4]. DD may affect parental functioning and management of their child’s T1D, which in turn is linked to greater instability in glycemic control and increased risk of complications [7,8]. Given the pivotal role of parents, effective interventions targeting this group are essential [9,10].

Research investigating parent-focused interventions in T1D has grown steadily since the early 1990s. A 2019 meta-analysis identified 17 randomized controlled trials (RCTs) addressing this topic [11]. With regard to psychological endpoints, pooled analysis of relevant studies found significant lower stress levels for intervention participants compared to control participants [11]. Although no differences were found in depressive symptoms, anxiety, or confidence around self-management, when removing studies that were outliers [12] or grouping studies using the same assessment instruments [13,14], improvements in depressive symptoms and anxiety were observed in the intervention compared to control participants. Notably, only three studies evaluated digitally delivered interventions.

Since 2019, there has been a significant shift towards digital models for T1D self-management and/or psychosocial support. For example, among 162 parents of adolescents with T1D, Whittemore et al. [15] evaluated an interactive six-session, web-based intervention aimed at improving the parent–adolescent relationship, communication strategies, gradual transfer of self-management responsibilities, emotional experience, and self-care prioritization. At 6 months post-intervention, reductions in parenting stress were observed for the intervention group compared to the control group; however, no differences were found for other psychosocial outcomes (e.g., general stress, depressive, and anxiety symptoms). Another parent-targeted RCT examined an intervention delivered using the WeChat platform, an education module, and a health professional-led questionnaire [16]. At 6 months post-intervention, the intervention group reported a lower frequency of depression and anxiety symptoms and higher ratings for quality of life (QOL) dimensions compared to the control group. Finally, an evaluation of Sugarsquare, a web-based portal offering virtual peer support, T1D information, and a channel for parent–health provider communication, found no intervention–control group differences in parenting stress [17]. Not only does heterogeneity in curriculum structure (manualized versus question and answer), delivery timing (synchronous versus asynchronous), platform type (web-based portal, telephone-based, and mobile app), and interventionists (health professionals and peers) make comparisons across studies difficult, but it is equally challenging to determine which intervention component is most effective.

Given the emphasis on personalized treatment in the digital era, the optimal approach to mental health support should present a range of options with regard to the type, intensity, modality, and structure, allowing users to customize their own support model. Huddle4Parents is a 4-month digital intervention that involves monthly professional-led, pre-scheduled, and video-based sessions coupled with a 24/7 peer support texting platform (Signal). This intervention offers support with different options as it is accessed synchronously and/or asynchronously, delivered by a clinical psychologist and/or a community of parent peers, hosted on a group-based face-to-face video and/or SMS texting platforms, organized around a semi-structured yet flexible curriculum (i.e., sessions can be modified based on parents’ concerns and priorities).

The objectives of this proof-of-concept study are two-fold: (1) to determine the feasibility, acceptability, and potential health impact of the 4-month Huddle4Parents intervention; and (2) to explore the core concerns and issues parents express when seeking support (from the peer texting platform).

## 2. Materials and Methods

### 2.1. Participants

This study was approved by the Behavioral Research Ethics Board at the University of British Columbia (H21-00226). We employed multiple strategies to recruit parents of children ages 5 to 9 living with T1D from three pediatric diabetes clinics in British Columbia (BC). At one site, we emailed an invitation letter and link to an online recruitment form to parents/families who previously consented to be contacted for research. At the other two sites, we posted flyers that included a study website link or QR code to access an online recruitment form. Those who completed the online form were contacted by research staff to provide more details about this study and facilitate the informed consent process.

Parents were eligible if they spoke English and had access to the internet and Zoom. Parents provided informed consent and completed baseline and 4-month surveys using REDCap electronic data capture tools [18,19]. Upon completion of each survey, participants received a USD 10 gift card.

### 2.2. Intervention

The intervention was held over 4 months and included two components:

(i) Huddles: synchronous virtual sessions (90 min) hosted on Zoom. Four monthly sessions were facilitated by a registered psychologist and a young adult living with T1D who was trained by the former. All sessions started with a review of community agreed-upon guidelines of conduct. The first Huddle invited parents to share their child’s diagnosis story. The second Huddle taught basic coping strategies and asked parents to describe one thing they were doing well and what type of support they needed. The third and last Huddles focused on parent-selected topics (e.g., school-based diabetes support, resilience).
(ii) 24/7 Peer Support Room: an asynchronous texting group hosted on the messaging platform, Signal. It provided a safe space for parents to pose questions, chat, seek advice, and build rapport with others in real-time. A registered psychologist and nurse educator monitored exchanges, and young adults with T1D who were part of the intervention team (*n* = 6) provided their perspective if requested by parents.

### 2.3. Measures

(i) The Sociodemographic Survey is a 12-item questionnaire (administered only at pre-intervention) that assesses a child and parent’s genders, ethnicity, education, household income, diabetes management, and insurance coverage.
(ii) Feasibility is defined as the ability to recruit *n* = 20 parents and retain at least 75% of the sample. Acceptability is defined as receiving program satisfaction ratings of “good” to “very good”.
(iii) Parent Version of Problem Areas in Diabetes-Child (P-PAID-C) is a 16-item validated questionnaire that assesses DD, emotional burden, and child regimen-specific distress in parents of children with T1D ages 8 to 12 [8].
(iv) Type 1 Diabetes and Life (T1DAL)-Parent is a 23- or 27-item (depending on age of the child) validated questionnaire that assesses diabetes-specific quality of life (QOL) [20]. Perceived Support Survey: a 6-item adapted questionnaire that assesses the amount of and satisfaction with support received from family, healthcare team, and other parents [21].

### 2.4. Data Analyses

Descriptive analyses were conducted on the demographics of the study participants and the outcome measures pre- and post-intervention. Paired *t*-tests were conducted for each outcome measure to determine whether the outcome measure changed as a result of the intervention.

A thematic analysis was conducted with the text exchanges in the Signal-based 24/7 peer support room. The research assistant (NS) documented the number of texts posted during the 4-month intervention and identified major themes and subthemes emerging from the exchanges. The research assistant also counted the number of messages that were classified under each theme. Messages that had multiple topics were counted separately. Images sent were counted separately.

## 3. Results

Of the 18 parents recruited, three dropped out prior to the start of this study. Two participants reported being “too busy”, while the third did not provide a reason. Table 1 presents sociodemographic characteristics of parents and their children at baseline. Mothers comprised 86.7% (*n* = 13) of the sample; 73.3% (*n* = 11) had at least a college degree; 53.8% (*n* = 7) reported an annual income of greater than $100,000. All parents (*n* = 15) identified as White. Of the children whose parents completed the study, 66.7% (*n* = 10) were between the ages of 8–9 years; all identified as White; 66.7% were male (*n* = 10); and 66.7% (*n* = 10) reported at least one sibling.

Table 2 summarizes the diabetes treatment, access to a private health plan, and diabetes technology use for children of participating parents. Sixty percent (*n* = 9) were on insulin pump therapy, and all (*n* = 15) used sensors.

No significant differences were seen between baseline and post-intervention in DD (P-PAID-C, *p* = 0.867), diabetes-specific quality of life (*p* = 0.133), and perceived social support (*p* = 0.436).

### 3.1. Feasibility

Our goal was to recruit 20 parents. We recruited 18 who provided consent. Of the 18 parents, 15 attended at least one Huddle and posted on the 24/7 peer support room. Attendance for the first two Huddles was 100% and 66.7% and 73.3% for the third and fourth Huddles, respectively. The median number of Huddles attended was 3.5. For the 24/7 peer support room, 15 parents were added to the Signal chat group and sent at least one message. Retention rate was 83%.

### 3.2. Acceptability

Between Huddle 1 to 4, 1084 messages were exchanged by the 15 parents. Parents completed a post-study satisfaction survey (*n* = 13), where they rated and provided feedback on the 24/7 peer support room and Huddles. Participants reported a high satisfaction for both, with 100% indicating that they would “definitely” or “probably yes” recommend both to others with children ages 5 to 9 living with T1D and rated the topics, facilitator, and overall Huddle as “good”, “very good”, and “excellent”.

### 3.3. Analysis of Posts from the 24/7 Peer Support Room

Of the 1084, 195 posts were categorized as “miscellaneous” (e.g., emojis, introductions, and health provider interjections) and omitted from the analysis. Table 3 presents the four major themes and associated subthemes, as well as the number of posts and examples of text exchanges for each of the categories. Theme one highlighted technology and devices and included two subthemes: (1a) seeking and providing advice, education, and information; and (1b) expressing fear, anxiety, and uncertainty. Theme two highlighted emotional support and included three subthemes: (2a) demonstrating empathy and understanding; (2b) checking in with the T1D parent community; and (2c) providing reassurance and motivation. Theme three highlighted T1D management-related tips and strategies and included two subthemes: (3a) generating and exchanging resources; and (3b) trouble-shooting, normalizing, and validating. Theme four highlighted T1D management in the school setting and included two subthemes: (4a) discussing nursing support services, educational assistants, and other resources; and (4b) sharing concerns and frustrations. It should be noted that in BC, nursing support services refers to the organization responsible for coordinating diabetes care in schools in BC.

## 4. Discussion

To our knowledge, Huddle4Parents is the first digital support intervention targeting parents of children with T1D that offers flexible support that is pre-scheduled and/or “in real time”, delivered by a mental health professional and peers, organized around a curriculum modifiable to parent priorities, and conducted via video and/or via text. Findings suggest that the intervention is feasible based on recruitment (18 out of 20 expected participants), participation (median attendance 3.5 out of 4 Huddles), and attrition rate (17%) and acceptable as demonstrated by engagement level and satisfaction ratings. Based on the texts posted and messages exchanged in the 24/7 peer support room, parents’ greatest support needs pertain to T1D technology and devices, emotional encouragement and assistance, T1D management in the school setting, and management-related tips and strategies. It should be noted that changes were not observed for DD or other psychosocial outcomes in this proof-of-concept study.

Our results are consistent with the findings of Sugarsquare, which was also deemed feasible and acceptable based on enrollment, attrition, and engagement statistics but showed no between-group differences in the primary psychosocial outcome (parenting stress) [17]. However, according to an evaluation of CARES, a telehealth group-based DD intervention that was offered to 41 parents of children with T1D ages 5–12, not only were the metrics for feasibility (20–25% attrition rate; 96–98% attendance rate) and acceptability (89 to 91% satisfaction rate) met, but this intervention also was associated with lower levels of DD and depressive symptoms at post-intervention and 3-month follow-up [22]. Notwithstanding the variability in intervention design (e.g., specific platform used, curriculum structure followed, and timing and intensity of support offered), it would appear that digital support models, in general, are viable and appealing to the T1D parent community. Although more large-scale and fully powered RCTs are needed to evaluate the impact of these interventions on mental health outcomes.

The 1084 posts by parents in the 24/7 peer support room across the 4-month intervention underscore the tremendous need and value of peer support. According to a scoping review on T1D caregiver burden, support from healthcare professionals may not be sufficient, as healthcare professionals have not experienced the same daily stigma, fear, and frustration as parents, nor do they have the availability to respond to parents’ concerns in “real-time” [23]. As such, peer support, especially those from online sources (e.g., T1D-specific Facebook groups or discussion boards), offers parents reassurance, validation, and normalization [23]. Not surprisingly, in several qualitative studies, parents have identified peer support as a critical component for digital support platforms [24,25,26,27]. These interventions, however, may not be ideally suited for all users. For instance, Rankin et al. [28] found that, during the critical time period following initial T1D diagnosis, some parents felt overwhelmed and, thus, not open to receiving support from other peers. Other parents expressed discomfort when receiving peer-initiated advice online that conflicted with physician recommendations. Therefore, to be most effective, peer support models should be designed to accommodate the unique needs and emotional journey of each parent as well as to safeguard users from giving or receiving medical/clinical information.

While the core themes extracted from the 24/7 peer support room (T1D technology, emotional support, management in the school setting, tips and strategies) have been cited in other similar studies [23,29,30,31], further in-depth analysis uncovered an additional finding. Specifically, embedded within each core theme was an emotion-focused subtheme. For example, “T1D technology and devices” encompassed two subthemes—one subtheme “seeking and giving advice, education, and information” reflected informational support needs, and the other subtheme “expressing fear, anxiety, and uncertainty” reflected emotional support needs. This dual dimension of support (pragmatic and emotive) also emerged for two other core themes: “T1D management in school” and “management-related tips and strategies”. It would appear that, for T1D parents, mental health is inextricably linked with self-management, and this relationship is likely bi-directional. While this association between mental health outcomes (e.g., DD, depressive symptoms) and self-management (e.g., medication adherence, blood sugar monitoring) has been reported in quantitative studies of the T1D population [4,5,7,9,10], this is one of the few studies that have highlighted this relationship using qualitative data. Clinical implications of these findings suggest that rather than compartmentalize mental health as a “separate topic” or exclude it altogether, diabetes care models may consider integrating these issues more seamlessly into the curriculum, emulating how it is experienced in real life.

## 5. Limitations

This study is not without limitations. First, this proof-of-concept investigation recruited a small sample size and was not powered to demonstrate pre-post changes in mental health outcomes. However, we did demonstrate the feasibility of collecting the relevant outcome measures and acceptability of the intervention itself. Second, our cohort was comprised largely of white and female parents; therefore, results are not generalizable to the larger T1D parent community. Finally, we did not measure diabetes-related outcomes in children. Given the research linking parents’ mental health functioning to glycemic outcomes for T1D children [4,32], personalized digital support interventions such as Huddle4Parents may have the potential to produce indirect diabetes-related health benefits for T1D children. Future RCTs recruiting a larger and more sociodemographically diverse population will better address these questions.

## 6. Conclusions

Caregivers, especially parents of young children with T1D, are affected by the ongoing stress and burden of managing diabetes. While research indicates that digital support models are appealing to the T1D parent community, these interventions vary in who, what, when, and how support is delivered. In the era of personalized medicine, offering parents as many options for seeking and securing support will likely increase engagement. Ultimately, the more our interventions can respond to the unique and evolving support needs of each individual parent, the better our mental health outcomes will be.

## Figures and Tables

**Table 1:** Demographic characteristics.

	N	%
Child’s Age group		
<8 years old	5	33.3
8–9 years old	10	66.7
Child’s Ethnicity		
White	15	100
Child’s Gender		
Male	10	66.7
Female	5	33.3
Child’s Number of Siblings		
0	5	33.3
1	7	46.7
2	3	20.0
Relationship to Child		
Father	2	13.3
Mother	13	86.7
Parent’s Ethnicity		
White	15	100
Parent’s Education (*n* = 15)		
High School Graduate or Some College	4	26.7
College Graduate	6	40.0
Graduate/Professional Degree	5	33.3
Partner of Parent’s Education (*n* = 15)		
High School Graduate or Some College	7	46.7
College Graduate	3	20.0
Graduate/Professional Degree	4	26.7
N/A	1	6.6
Location of Diabetes Care (*n* = 15)		
Children’s Hospital Diabetes Clinic	9	60.0
Community Based Diabetes Clinic	6	40.0
Annual Household Income (*n* = 13, 2 decided not to disclose)		
<$49,999	2	15.4
$50,000–69,999	0	0.0
$70,000–99,999	4	30.8
>$100,000	7	53.8

**Table 2:** Summary of child’s diabetes treatment, technology use, and health coverage.

	N	%
Child’s current diabetes treatment (*n* = 15)		
Multiple insulin injections per day (before all meals and large snacks)	6	40.0
Continuous subcutaneous insulin infusion (insulin pump)	9	60.0
Private Health Insurance Available to Child (*n* = 15)		
Yes	10	66.7
No	5	33.3
Talk to Health Plan Carrier about T1D-Related Treatment and Tech (*n* = 10)		
0–1 h/month	5	50.0
1–2 h/month	2	20.0
3 or more hours/month	3	30.0
Insulin Coverage by Health Plan (*n* = 10, health plan available to child)		
No Coverage	1	10.0
Partial Coverage	3	30.0
Full Coverage	6	60.0
Pump Coverage by Health Plan (*n* = 6, using pump and health plan available to child)		
No Coverage	0	0
Partial Coverage	1	16.7
Full Coverage	5	83.3
Child Sensor Use (*n* = 15)		
Yes	15	100
No	0	0
Type of Sensor used by Child (*n* = 15)		
Flash Glucose Monitor (FGM)	1	6.7
Continuous Glucose Monitor (CGM)	14	93.3
Sensor Coverage by Health Plan (*n* = 10, health plan available to child)		
No Coverage	1	10.0
Partial Coverage	2	20.0
Full Coverage	7	70.0
Child Counseling Services Coverage by Health Plan (*n* = 10, health plan available to child)		
Yes	6	60.0
No	4	40.0
Parent Counseling Services Coverage by Health Plan (*n* = 10, health plan available)		
Yes	9	90.0
No	1	10.0

**Table 3:** The 24/7 peer support room themes and subthemes (889 posts analyzed).

Theme 1. Technology and devices (253 posts)			
1a. Seeking and providing advice, education and information		1b. Expressing fear, anxiety, and uncertainty.	
(Informational support)		(Emotional Support)	
Parent A: “Can some patient tech savvy person please tell me how to see [child’s name] BG numbers from her DexCom on my Garmin Forerunner 35 watch? She sees her numbers on the DexCom app on an iPhone, and we have the DexCom Follow app on our phones, but I would really like to see them on my watch rather than having to look at my phone all the time.”		Parent A: “Technology has always kind of overwhelmed me. We have had so much happen the last 2 years, it seemed every [time] I was ready to take it on it would be another setback. We got a sensor in the summer and I feel like we have it down pat. I just need to take the steps to start and get it going.”	
Parent B: “We aren’t on Dexcom anymore but I used to use an app called sugarmate-mind you this was to see it on my Apple Watch....wonder if it would work with yours....There is also Nightscout-several different options with nightscout-I’ll try and see if I can find your specific watch on a list.”		Parent B: “I was also terrified at the start. Just like we had gotten ‘comfortable’ with things and then back to the unknown. [It] was hard. When you transition to a pump, you usually have access to the pump trainer for an entire month. Way more support when transitioning to a pump, unlike the sensor.”	
Theme 2. Emotional support (224 posts)			
2a. Demonstrating empathy and understanding	2b. Checking-in with the T1D parent community		2c. Providing reassurance and motivation
(Emotional support)	(Emotional/Community support)		(Affirmational support)
Parent A: “Is anyone up? This pumping is stressful! I think I have to change the pod out. He’s quite high and the correction isn’t helping. I was told the best thing is to change the pod, but holy hell I’m anxious. I don’t know whether to leave him sleeping and trying to change it while he sleeps or wake him. I hate diabetes!”	Parent A: “Good morning, happy Friday! How’s everyone doing today?”		Parent A: “You truly are a warrior, and I hope you feel the support around you within this group”
Parent B: “Oh man! I can imagine how stressful that is. I’m sorry you are having to deal with that.”	Parent B:” Our night sucked over here! The sensor updated for a couple hours when I decided that it would probably end up saying change, so I pulled it and started a new one. The first day requires so many calibrations the first few hours, so not much sleep. Love the sensor system, hate when the sensor messes up. Common for us around day 5.”		Parent B: “As a silver lining, your overnight number look spectacular!”
Theme 3. T1D management in the school setting (204 posts)			
3a. Discussing nursing support services, educational assistants, and other resources		3b. Trouble-shooting, normalizing and validating	
(Informational support)		(Emotional/community support)	
Parent A: “After dealing with NSS and care plans for the last 4 years ([child’s name] in grade 3 now-diagnosed at age 3) I have come [to] learn that there is a lot of room [for] improvement with care plans and EA support at school. If you guys are anything like me the school day is a huge contributing factor to my own personal stress.”		Parent A: “And some days he has gym half an hour after lunch, there have been days where he says in the classroom with his Educational Assistant because he just isn’t willing to eat more, but it’s too close to low for them to allow him to go do gym without a snack! It’s so challenging.”	
Parent B: “Our NSS gal was fierce and awesome. She hand picked the EA she wanted for [child’s name] as had prior experience with type 1 students and had a flexible attitude that matched our way of managing diabetes.”		Parent B: “My son has reverse lunch as well, and quite often needs snack before going out to play, it’s such a shit schedule for a T1D, but his school didn’t have flexibility of changing it unfortunately.”	
Theme 4. T1D-related tips and strategies (208 posts)			
4a. Generating and exchanging resources		4b. Sharing concerns, frustration, and experiences	
(Tangible support)		(Emotional/community support)	
Parent A: “This doesn’t work for everyone but we have had a ‘diabetes backpack’ from day one loaded with anything we could need. There are snacks, fast sugars, everything for full site changes, including extra infusion sets or pods, insulin, sensors, alcohol wipes, tapes, batteries, charging cables, insulin pens-you name it.”		Parent A: “On the sensor topic, anyone have a sensor get stuck and not release after pressing the orange button? We’ve had it twice. It. Was. Awful. Terrifying for my daughter. She refused to try the sensor again for months.”	
Parent B: “The Juicebox podcast is the best!! They have a great episode about changing from MDI to pumps that you can listen to.”		Parent B: “Just found this after your post: [Image with text: “Is anyone aware that if the sensor doesn’t release from the inserter, you can poke this small hole with safety tab to release it?”]. It was originally on the Beyond Type 1 forum.”	
Parent C: “[Child’s name] has anxiety that comes out as a need to control everything. It’s up and down like a roller coaster. Although it wasn’t a god fit for [child’s name], the website “Go Zen” is a fantastic resource. Downside is it’s expensive. They have tons of different animated modules about anxiety, anger, stress, perfectionism, etc.”			

## Data Availability

The raw data supporting the conclusions of this article will be made available by the authors on request due to privacy restrictions.
